# The effect of a spinal thrust manipulation’s audible pop on brain wave activity: a quasi-experimental repeated measure design

**DOI:** 10.7717/peerj.17622

**Published:** 2024-06-28

**Authors:** Rob Sillevis, Joshua Unum, Valerie Weiss, Eric Shamus, Francisco Selva-Sarzo

**Affiliations:** 1 Department of Rehabilitation Sciences, Florida Gulf Coast University, Fort Myers, FL, USA; 2 Physiotherapy, Universitat de Valencia, Valencia, Spain

**Keywords:** Audible pop, Thrust manipulation, Brainwave

## Abstract

**Introduction:**

High velocity thrust manipulation is commonly used when managing joint dysfunctions. Often, these thrust maneuvers will elicit an audible pop. It has been unclear what conclusively causes this audible sound and its clinical meaningfulness. This study sought to identify the effect of the audible pop on brainwave activity directly following a prone T7 thrust manipulation in asymptomatic/healthy subjects.

**Methods:**

This was a quasi-experimental repeated measure study design in which 57 subjects completed the study protocol. Brain wave activity was measured with the Emotiv EPOC+, which collects data with a frequency of 128 HZ and has 14 electrodes. Testing was performed in a controlled environment with minimal electrical interference (as measured with a Gauss meter), temperature variance, lighting variance, sound pollution, and other variable changes that could have influenced or interfered with pure EEG data acquisition. After accommodation each subject underwent a prone T7 posterior-anterior thrust manipulation. Immediately after the thrust manipulation the brainwave activity was measured for 10 seconds.

**Results:**

The non-audible group (*N* = 20) consisted of 55% males, and the audible group (*N* = 37) consisted of 43% males. The non-audible group EEG data revealed a significant change in brain wave activity under some of the electrodes in the frontal, parietal, and the occipital lobes. In the audible group, there was a significant change in brain wave activity under all electrodes in the frontal lobes, the parietal lobe, and the occipital lobes but not the temporal lobes.

**Conclusion:**

The audible sounds caused by a thoracic high velocity thrust manipulation did not affect the activity in the audible centers in the temporal brain region. The results support the hypothesis that thrust manipulation with or without audible sound results in a generalized relaxation immediately following the manipulation. The absence of a significant difference in brainwave activity in the frontal lobe in this study might indicate that the audible pop does not produce a “placebo” mechanism.

## Introduction

Joint manipulation is commonly used by a variety of healthcare practitioners when managing musculoskeletal disorders (Moorman & Newell, 2022). Joint manipulation has been defined as a maneuver in which the joint is passively moved through various ranges. This movement can vary in speed and includes the small amplitude high-velocity thrust (HVLA) manipulation (Sillevis et al., 2010). HVLA manipulation appears to be beneficial for some musculoskeletal conditions, and it is reported to reduce the perception of pain, improve mobility, and reduce self-perceived disability (Evans & Lucas, 2010). During an HVLA manipulation, a rapid stretch of the periarticular structures occurs without lasting change in the articular separation (Evans & Lucas, 2010). This separation often results in a popping sensation, sometimes just felt by the patient and clinician, and sometimes it results in an audible popping sound perceived by both patient and clinician (Cramer et al., 2011; Evans & Lucas, 2010). The audible pop is often thought of as a necessary criterion when defining a successful HVLA manipulation (Evans & Lucas, 2010). Additionally, it has been suggested that the combination of an audible pop with therapeutic touch by the clinician could result in alteration of ascending and descending sensory neural pathway activity and thus contribute to the successful perception of an HVLA manipulation (Herzog, Kats & Symons, 2001).

Despite this, the exact mechanism by which the audible pop is produced remains elusive and continues to be debated (Chandran Suja & Barakat, 2018). Because the audible pop only occurs during the HVLA manipulation of synovial joints, it infers a causative relationship between the joint capsule and synovia (Bereznick et al., 2008). Currently, the most accepted theories explaining the audible pop are the cavitational collapse and tribonucleation theories (Bereznick et al., 2008; Kawchuk et al., 2015). The cavitational collapse theory hypothesizes that the rapid reduction in intraarticular joint pressure, caused by the HVLA manipulation, releasing nitrogen gas from the synovial fluid, causing the audible pop (Sillevis & Cleland, 2011; Moorman & Newell, 2022). Currently, the acoustic signature of cavitation bubble collapse is consistent with experimentally observed sounds, thus lending support for cavitation bubble collapse as a potential source of the sound (Chandran Suja & Barakat, 2018). The tribonucleation theory hypothesizes that a rapid joint capsule elongation causes a decrease in internal joint pressure (Bereznick et al., 2008; Kawchuk et al., 2015). However, this change in pressure causes a bowing of the edges of the joint cartilage away from the articular bone. This bowing of the cartilage is immediately followed by the release of carbon dioxide from the synovial fluid. This release of carbon dioxide, in turn, will normalize the intra-articular pressure, and the joint cartilage will rapidly slap back against the cortical bone surface, creating the audible pop (Bereznick et al., 2008).

Subjects undergoing spinal manipulation and clinicians continue to correlate the presence of the audible pop to the success of an HVLA manipulation (Mourad et al., 2022). Anecdotally, patients believe that an HVLA manipulation producing an audible pop is more effective than a manipulation that does not produce an audible sound (Cramer et al., 2011; Evans & Lucas, 2010). The relationship between patient expectations (positive or negative) and treatment outcomes has been previously demonstrated (Bishop, Bialosky & Cleland, 2011). Based on this relationship, it is necessary to further evaluate if the audible pop affects the central nervous system and determine if there is any therapeutic value to the audible pop. A way to measure central nervous system activity is with the use of an electroencephalograph (EEG) (Light et al., 2010). Despite its inherent limitations, the EEG is considered one of the few real-time measures directly reflecting neural activity in the brain (Roy et al., 2019). The EEG measures electrical potential differences generated in the cortical layers of the brain (Roy et al., 2019). The potential differences are generated by both excitatory and inhibitory synaptic potentials, which create electrical currents that flow through the extracellular spaces of the brain (Roy et al., 2019). The total amount of brainwave activity is reflected by the frequency of signals measured on the scalp in Hertz (Hz) (Light et al., 2010). These frequencies are typically classified into five waves: Delta, Theta, Alpha, Beta, and Gamma waves (Light et al., 2010; Williams et al., 2020). Each type of wave represents different correlated brain activity. Delta waves range from .1 to 4 Hz and are generally present in stages 3-4 of sleep, however, they are abnormal in waking adults (Williams et al., 2020). Theta waves range from 4 to 8 Hz and are related to subconscious activity, often observed in relaxation, associated with the production of cortical hormones like serotonin, and are related to pain modulation (Kumar & Bhuvaneswari, 2012). Alpha waves range from 8 to 13 Hz; they are most commonly present in relaxed adults with their eyes closed (Kumar & Bhuvaneswari, 2012). Alpha waves are primarily present in the occipital and parietal regions and they represent activity of the brain’s white matter (Kumar & Bhuvaneswari, 2012). Beta waves range from 13 to 30 Hz and correspond with certain behaviors like talking, decision-making, or the use of judgment (Kumar & Bhuvaneswari, 2012). Beta waves are related to our sensory intake, especially in what we see, touch, hear, smell, and taste (Kumar & Bhuvaneswari, 2012). Beta waves are usually found bilaterally in the frontal and parietal lobes and are associated with cortisol (Kumar & Bhuvaneswari, 2012). Finally, gamma waves range from 31 to 150 Hz (Kumar & Bhuvaneswari, 2012). These waves are associated with perception and consciousness and indicate alertness and the intake of sensory information (Kumar & Bhuvaneswari, 2012).

Any change in human perception would be expressed by a change in neural activity in the brain, and thus the resulting in a change in EEG signaling. Hence, if HVLA manipulations produce an audible pop, this should at least result in an immediate alteration in brainwave activity in the brain’s auditory centers in the temporal lobe (Sparks et al., 2017). By identifying the presence or absence of differences in EEG brain activity in healthy subjects who produce an audible pop while undergoing an HVLA manipulation, we might better understanding the impact that the audible pop on brainwave activity.

The clinical meaningfulness of the audible pop remains unclear. Flynn et al. (2003) demonstrated no correlation between the audible pop and decreased post-manipulation pain levels. This finding concurs with Sillevis & Cleland (2011), who demonstrated that the audible pop during an HVLA thoracic manipulation did not cause any significant change in autonomic nervous system activity, nor a reduction in pain (Sillevis & Cleland, 2011). The lack of correlation between the presence of an audible pop and pain reduction was further supported by the findings of Cleland et al. (2007) and Moorman & Newell (2022). In summary, there is currently no evidence that the audible pop impacts clinical (*e.g.*, pain and self-reported disability) or physiological (*e.g.*, inflammatory markers and autonomic nervous system) outcomes (Cleland et al., 2007; Sillevis & Cleland, 2011; Moorman & Newell, 2022; Flynn et al., 2003). Therefore, this study aimed to identify and quantify any changes that were present in the brain EEG signals in the presence or absence of an audible pop directly following a posterior to anterior (PA) HVLA manipulation at the thoracic seven vertebrae (T7).

## Material and Methods

### Study design

This quasi-experimental study used a method of convenience sampling with a within-subject repeated measure design. Subjects were recruited for three months in the Fall of 2020 using a flyer and word of mouth. This study received institutional review board (IRB# 2020-64) approval from Florida Gulf Coast University; additionally, this study was registered at Clinical Trial.gov with ID# NCT04542707. All subjects provided written consent prior to participating in the study.

### Subjects

A total of 57 asymptomatic healthy subjects were recruited. All available subjects were screened for the eligibility criteria. To participate, all subjects had to be between the ages of 18 and 65 and be able to read English so proper written consent could be given. Exclusion criteria were thoracic spine injuries, with non-specific spinal pain within six months of the experiment, naïve to spinal manipulation, osteopenia/ osteoporosis, cancer, or any other conditions or medications that would lead to skeletal compromise (such as osteogenesis imperfecta, chemotherapy, or long-term high-dose corticosteroid usage). Additional exclusion criteria were a positive history of concussion, traumatic brain injury (TBI), or brain damage due to the potential for atypical neural signaling because of neuroplastic changes after the injury.

### Study protocol

Testing was performed in a controlled environment with minimal electrical interference (as measured with a Gauss meter), temperature variance, lighting variance, sound pollution, and other variable changes that could have influenced or interfered with pure EEG data acquisition. After providing written consent, each subject was brought into the testing room. Subjects removed their shirts and were seated for to apply the Emotive EPOC+ headset. The Emotiv Pro software provided an assessment of connectivity to ensure quality data transmission.

After the headset was positioned correctly on the subject’s head, the second researcher identified the T7 vertebrae by palpation. To consistently identify the T7 level for this study, each subject was tested by the same researcher. In all patients, C7 was identified as the vertebrae that has the largest spinous process, and C6 was identified as that spinous process that would relatively disappear upon extension motion of the cervical spine (Brismee et al., 2006; Vleeming, Winkel & Meijer, 1984). Passive neck flexion was used to identify inter-segmental motion to determine the T7 level (Paris, 2000). A clear mark was placed on the skin identifying the T7 spinous process.

Next, the subject was placed in the prone position on a treatment table. One must consider that both the position on the table and the clinical touch during the HVLA results in sensory perception by the subject and thus may correspond to a change in brainwave activity. Touch has been previously shown to improve one’s subjective assessment of physical and psychological function. Physical contact by itself can provide a placebo benefit to patients for reducing both anxiety and pain (Morita et al., 2018). Morita et al. (2018) previously demonstrated that physical touch in the interscapular region results in an altered brainwave activity pattern in the primary somatosensory cortex (supramarginal and postcentral gyrus).

Our subjects were instructed to relax, stay still, and close their eyes to avoid any potential artifacts caused by ocular or muscular activity (Kumar & Bhuvaneswari, 2012; Light et al., 2010). As previously described, the subject was placed in the prone position while the second researcher positioned his hands to perform a non-specific HVLA of T7. The subject and clinician remained in this pre-intervention hold position for 15 s to normalize brain activity, decreasing the direct effect of touch. A 10-second pre-intervention EEG measure was taken. This choice was made to use 10-seconds based on trial before this study. Since brain wave activity changes so rapidly a significant effect of the manipulation on any of the EEG channels could be missed with prolonged measuring.

Next, the subject underwent the HVLA at the patient’s apex of exhalation. Immediately following the performance of the HVLA, the post-intervention position was maintained for 15 s with the clinician’s hands in the same position, after which a 10 s duration post-intervention EEG was recorded. Hands were kept in position after the HVLA was performed to maintain identical external sensory stimuli to the brain before and after the HVLA, thus reducing the opportunity to measure the physiological artifacts unrelated to this experiment. Following the post-intervention measure, the therapist noted whether the participant or therapist heard an audible pop directly after the HVLA.

### Manipulation

**Figure 1:**
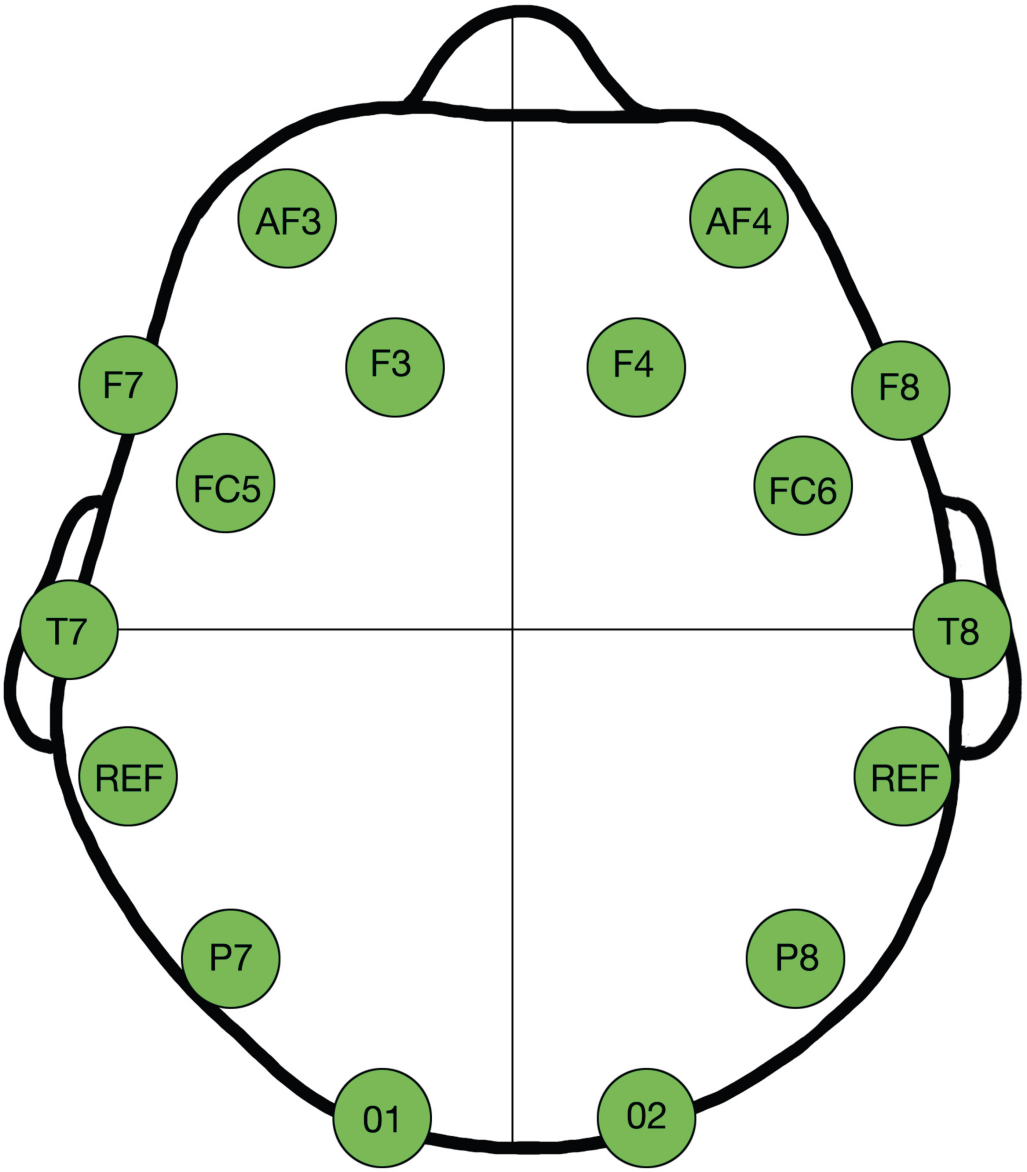
Location of the 14 lead Emotiv Epoc+.

Each subject underwent a PA HVLA targeting the seventh thoracic vertebra while in the prone position. All manipulations were performed by a single person trained in manual therapy with more than five years of clinical experience. For this HVLA to be successful, the clinician placed each hand on opposite sides of the spinous process. The hypothenar of the near hand was placed on the transverse process of the target segment T7, and the thenar eminence of the far hand was placed on the contralateral transverse process of the adjacent segment (Fig. 1). Both arms applied an initial anterior-directed force to take up tissue slack, which was performed during a patient’s exhalation. Directly following this, during the patient’s second exhalation, the HVLA was carried out, a maneuver described by Hartman, (1997).

### Brainwave data

The headset was positioned on the subject’s head by a first researcher with more 50 h of experience with EPOC+ headset placement. He was blinded to the fact that the HVLA resulted in either an audible pop or not. The use of EEG measuring neural activity in the brain allows for identifying changes in brain wave activity compared to baseline measuring for each wave under each electrode. Since EEG measures are done in real-time, physiological artifacts can be generated by the subjects, which could create such brainwave spikes leading to measurement error. To minimize such an effect on the measurements used in this study, the brainwave measurement protocol was time-based, regardless of if they resulted in a spike of brain activity. The methodology chosen in this quasi-experimental study allowed for identification where in brain changes may occur and more specifically at what range they occur.

**Figure 2:**
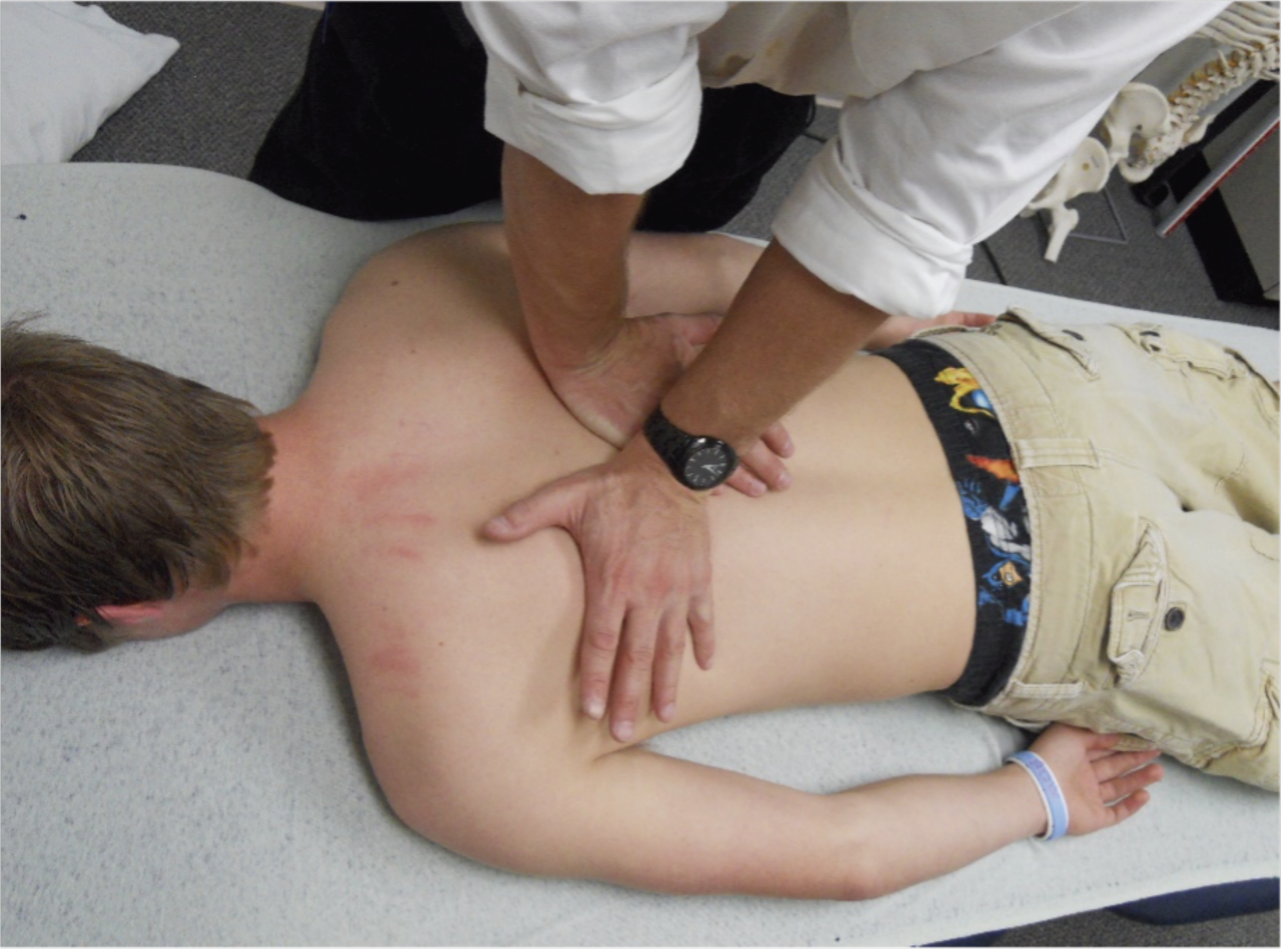
Manipulation technique: Prone posterior to anterior thoracic spine manipulation. (Person in the image is not study subject).

All EEG data were collected using the Bluetooth wireless Emotiv EPOC+ and the signal acquisition and processing Emotiv Pro software. The EPOC+ collects data with a frequency of 128 HZ and has 14 and two reference electrodes. The wireless Emotiv EPOC+ EEG system has been previously used and validated (Anderson et al., 2011; Blanco et al., 2019; Kotowski et al., 2018). The 14 electrodes were organized in such a way on the scalp that they approximate the location of AF3, AF4, F3, F4, F7, F8, FC5, FC6, T7, T8, P7, P8, O1, O2 of the international 10–20 system (Anderson et al., 2011; Blanco et al., 2019; Kotowski et al., 2018). Within this organization, as seen in Fig. 2, the AF3 and 4, F3 and 4, F7 and 8 electrodes represent the frontal lobe. The T7 and 8 electrodes represent the temporal lobe. The P7 and P8 electrodes represent the temporo-occipital area, and the O1 and O2 electrodes represent the occipital lobe. The EEG data from each electrode was directly recorded in the Emotive Pro program. Each electrode measures neural transmitter activity and thus voltages at the scalp within the Theta, Alpha, Beta, and Gamma wave bands.

### Statistics

Statistical analyses were performed using IBM’s SPSS, version 27.0, statistical software package (Armonk, NY, USA). All data were analyzed using a confidence interval of 95% and a significance level of 0.05. The brainwaves for each of the bands (Theta, Alpha, Beta, and Gamma waves) were analyzed for normal distribution using the Sharpo-Wilk test of normality, and all data were not normally distributed with *P* < 0.05; for that reason, the assumption for parametric statistics was not satisfied.

The subjects in this quasi-experimental study underwent a pre-post intervention repeated measure design. The data collected were analyzed using the Mann–Whitney U test to assess the difference between the pre-and post-intervention means of the five frequency bands for each electrode. This analysis was performed for the audible pop and the “non-audible pop” groups. Following this, the post-intervention data between both groups were analyzed using the Mann–Whitney U test to confirm or deny the presence of a significant change due to the audible pop. Additionally, the percentage of change for each frequency band in both groups was evaluated as this might shed light on the brain’s response based on the localization of observed changes.

## Results

All fifty-seven subjects completed the measurement and interventional protocol. There were 30 female and 27 male subjects. The non-audible group had 20 subjects (*N* = 20), consisting of 55% male and 45% females. The audible group had 37 subjects (*N* = 37), consisting of 43% male and 57% female subjects.

### Non-audible group

The Mann–Whitney test was used to determine the difference in the non-audible group between the pre- and post-HVLA manipulation effect on the different brain bands under each electrode (Table 1). There were a statistical significant difference (*p* < .05) between pre and post-intervention at the following electrodes: F8 electrode for the Beta-L (*p* = .040) and Theta (*p* = .028) band waves, at the AF3 electrode for the Theta (*p* = .002) and Beta-L (*p* = .023) band waves, at the F7 electrode for the Theta (*p* = .044) and Beta-L (*p* =.033) band waves, at the O1 electrode for the Beta-L (*p* = .004) and Beta-H (*p* = .004) band waves, at the P7 electrode for the Alpha (*p* = .017) waves, and P8 Beta-H (*p* = .000) band waves. In summary, this finding indicates that there was a significant change in brain wave activity in the frontal, parietal, and the occipital lobes.

**Table 1:** Pre-intervention *vs* post-intervention assessment for significant changes in the “no audible pop” group. Bold indicates the wave band and significant difference.

Band wave				
Significance Mann–Whitney U	**F8.Theta**	F8.Alpha	**F8.BetaL**	F8.BetaH	F8.Gamma
*P* value	.**028**	.370	.**040**	.575	.057
	**AF3.Theta**	AF3.Alpha	AF3.BetaL	AF3.BetaH	AF3.Gamma
*P* value	.**002**	.263	.**023**	.411	.073
	**F7.Theta**	F7.Alpha	**F7.BetaL**	F7.BetaH	F7.Gamma
*P* value	.**004**	.067	.**033**	.100	.093
	O1.Theta	O1.Alpha	**O2.BetaL**	**O2.BetaH**	O1.Gamma
*P* value	.079	.218	.**004**	.**004**	.218
	**P7.Theta**	P7.Alpha	P7.BetaL	**P7.BetaH**	P7.Gamma
*P* value	**.044**	**.017**	.709	**.204**	.575
	P8.Theta	P8.Alpha	P8.BetaL	**P8.BetaH**	P8.Gamma
*P* value	.135	.823	.455	.**000**	.940
	F4.Theta	F4.Alpha	F4.BetaL	F4.BetaH	F4.Gamma
*P* value	.601	.455	.526	.681	.709
	FC6.Theta	FC6.Alpha	FC6.BetaL	FC6.BetaH	FC6.Gamma
*P* value	.204	.391	.191	.433	.062
	AF4.Theta	AF4.Alpha	AF4.BetaL	AF4.BetaH	AF4.Gamma
*P* value	.145	.654	.455	.575	.940
	F3.Theta	F3.Alpha	F3.BetaL	F3.BetaH	F3.Gamma
*P* value	.218	1.000	.502	.794	.411
	FC5.Alpha	FC5.Alpha	FC5.BetaL	FC5.BetaH	FC5.Gamma
*P* value	.881	.135	.057	.156	.052
	O2.Theta	O2.Alpha	O2.BetaL	O2.BetaH	O2.Gamma
*P* value	.167	.526	.391	.062	.073
	T7.Theta	T7.Alpha	T7.BetaL	T7.BetaH	T7.Gamma
*P* value	.911	.852	.940	.502	.601
	T8.Theta	T8.Alpha	T8.BetaL	T8.BetaH	T8.Gamma
*P* value	.627	.156	.911	.709	.681

### Audible group

The Mann–Whitney test was used to determine the difference in the audible group between the pre- and post-HVLA manipulation effect of the different brain bands under each electrode. There were statistical significant differences (*p* < .05) between pre and post intervention at the following electrodes: AF3 electrode for the Theta (*p* = .038), Beta-L (*p* = .000), Beta-H (*p* = .000), and Gamma (*p* = .000) band waves, the AF4 electrode for all band waves (*p* = .009), the F3 electrode for the Theta (*p* = .001), Beta-L (*p* = .001), Beta-H (*p* = .000), and Gamma (*p* = .000) band waves, the F4 electrode for the Theta (*p* = .005), Beta-L (*p* = .001), Beta-H (*p* = .000), and Gamma (*p* = .000) band waves, the F7 electrode for all band waves (*p* = .021), the F8 electrode for all band waves (*p* < .001), the FC5 electrode for all band (*p* = .008), the FC6 electrode for all band waves (*p* = .030), the O1 electrode for the Theta (*p* = .017), Beta-L (*p* = .011), Beta-H (*p* = .001), and Gamma (*p* = .001) band waves, the O2 electrode for the Theta (*p* = .013), Beta-L (*p* = .017), Beta-H (*p* = .001), and Gamma (*p* = .000) band waves, the P7 electrode for all band waves (*p* = .022), and the P8 electrode for the Theta (*p* = .010), Beta-L (*p* = .015), Beta-H (*p* = .012), and Gamma (*p* = .002) band waves (Table 2). In summary, this finding indicates a significant change in brain wave activity under all electrodes in the frontal lobes, the parietal lobe, and the occipital lobes but not the temporal lobes.

**Table 2:** Pre-intervention *vs* post-intervention assessment for significant changes in the audible pop group. Bold indicates the wave band and significant difference.

Band wave					
Significance Mann–Whitney U	**AF3.Theta**	AF3.Alpha	**AF3.BetaL**	**AF3.BetaH**	**AF3.Gamma**
*P* value	.**038**	.111	**.000**	**.000**	**.000**
	**AF4.Theta**	**AF4.Alpha**	**AF4.BetaL**	**AF4.BetaH**	**AF4.Gamma**
*P* value	**.008**	**.009**	**.002**	**.001**	**.000**
	**F3.Theta**	F3.Alpha	**F3.BetaL**	**F3.BetaH**	**F3.Gamma**
*P* value	**.001**	.099	**.001**	**.000**	**.000**
	**F4.Theta**	F4.Alpha	**F4.BetaL**	**F4.BetaH**	**F4.Gamma**
*P* value	**.005**	.118	**.001**	**.000**	**.000**
	**F7.Theta**	**F7.Alpha**	**F7.BetaL**	**F7.BetaH**	**F7.Gamma**
*P* value	**.002**	**.021**	**.001**	**.001**	**.000**
	**F8.Theta**	**F8.Alpha**	**F8.BetaL**	**F8.BetaH**	**F8.Gamma**
*P* value	**.000**	**.001**	**.001**	**.001**	**.000**
	**FC5.Theta**	**FC5.Alpha**	**FC5.BetaL**	**FC5.BetaH**	**FC5.Gamma**
*P* value	**.002**	**.008**	**.004**	**.000**	**.000**
	**FC6.Theta**	**FC6.Alpha**	**FC6.BetaL**	**FC6.BetaH**	**FC6.Gamma**
*P* value	**.005**	**.030**	**.015**	**.003**	**.000**
	**O1.Theta**	O1.Alpha	**O1.BetaL**	**O1.BetaH**	**O1.Gamma**
*P* value	**.017**	.145	**.011**	**.001**	**.001**
	**O2.Theta**	O2.Alpha	**O2.BetaL**	**O2.BetaH**	**O2.Gamma**
*P* value	**.013**	.689	**.017**	**.011**	**.000**
	**P7.Theta**	**P7.Alpha**	**P7.BetaL**	**P7.BetaH**	**P7.Gamma**
*P* value	**.011**	**.022**	**.001**	**.001**	**.002**
	**P8.Theta**	P8.Alpha	**P8.BetaL**	**P8.BetaH**	**P8.Gamma**
*P* value	**.010**	.111	**.015**	**.012**	**.002**
	T7.Theta	T7.Alpha	T7.BetaL	T7.BetaH	T7.Gamma
*P* value	.071	.354	.338	.316	.338
	T8.Theta	T8.Alpha	T8.BetaL	T8.BetaH	T8.Gamma
*P* value	.202	.551	.521	.656	.712

### Between-group comparison

**Figure 3:**
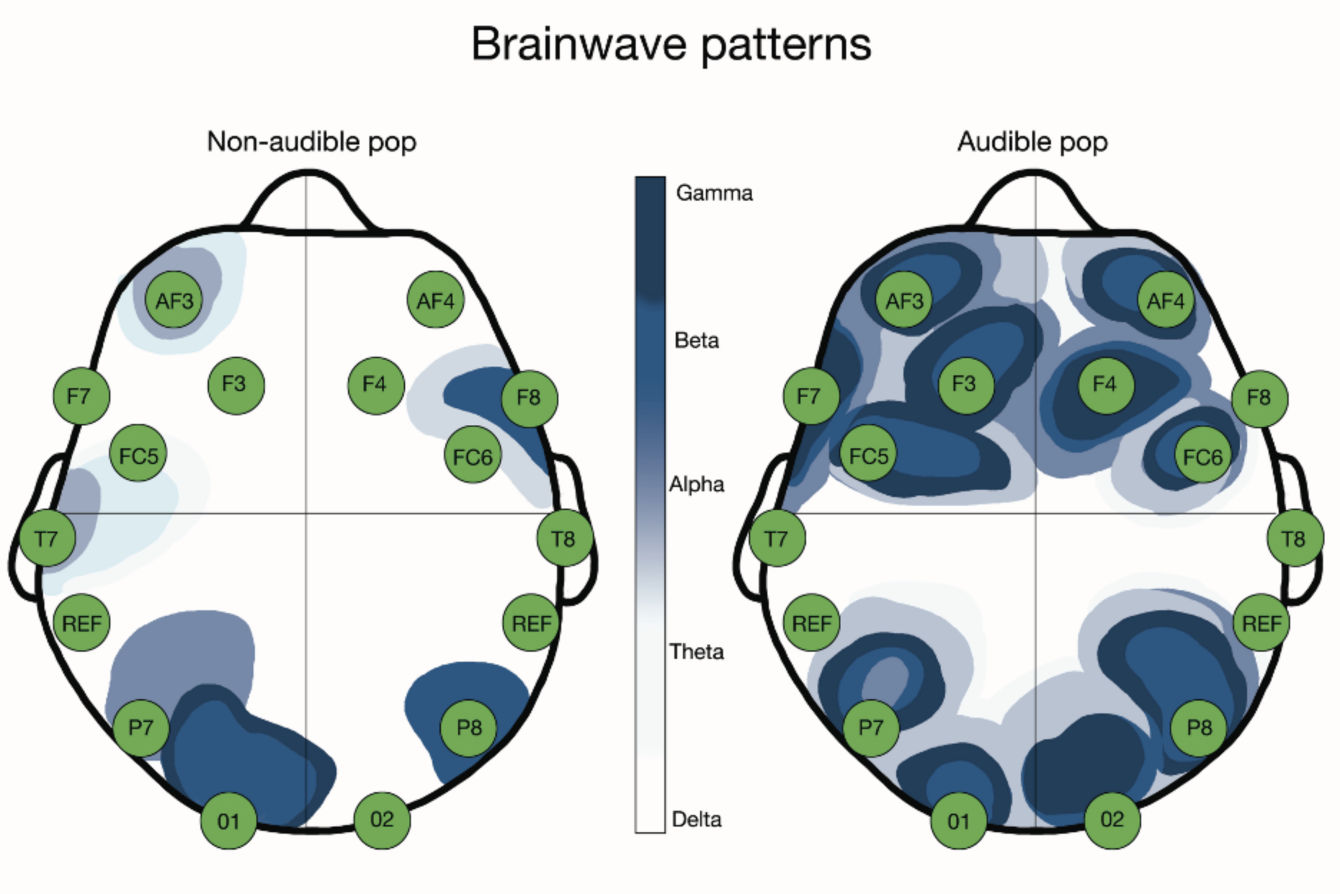
Post intervention significant changes in brainwave activity. Left side the activity in the non-audible group and on the right side the audible group. The green circles represent the 14 Emotiv electrodes.

The Mann–Whitney test was used to determine the difference between the audible and non-audible groups for the pre- and post-HVLA manipulation measures. There was no significant difference for the pre-intervention comparison (*P* > 0.05) between the groups. After the HVLA manipulation, there was a significant difference between the audible and non-audible groups under the P7 electrode for the Theta (*p* = .024) band wave, the O1 electrode for the Beta-H (*p* = .032) band wave, and the P8 electrode for the Beta-L (*p* = .048) band wave. Additionally, for each electrode and band wave the percentile changes of change in brain wave activity were identified (positive and negative changes) (Table 3 & Fig. 3).

**Table 3:** Post-intervention assessment of significance between audible pop *vs* “no audible pop” group. Bold indicates the wave band and significant difference.

**No audible pop % change**	Theta	Alpha	Beta-L	Beta-H	Gamma	**Audible pop % change**	Theta	Alpha	Beta-L	Beta-H	Gamma
F3	93%	12%	5%	2%	23%	F3	398%	74%	102%	87%	55%
F4	−2%	−10%	−22%	−16%	2%	F4	602%	173%	181%	196%	128%
AF3	261%	46%	41%	21%	55%	AF3	344%	150%	192%	125%	69%
AF4	−34%	−19%	−19%	−18%	22%	AF4	−24%	−32%	−38%	−49%	−34%
FC5	153%	27%	12%	13%	18%	FC5	454%	246%	214%	143%	89%
FC6	130%	49%	18%	-6%	23%	FC6	458%	129%	107%	87%	65%
F7	279%	49%	39%	32%	26%	F7	313%	178%	169%	106%	51%
F8	142%	33%	26%	13%	20%	F8	369%	117%	119%	83%	61%
P7	328%	76%	11%	−3%	−14%	P7	311%	167%	121%	154%	106%
P8	288%	128%	−17%	−8%	6%	P8	46%	20%	13%	−4%	19%
T7	−34%	−14%	−8%	−10%	−7%	T7	−95%	−85%	−78%	−78%	−69%
T8	4%	6%	−3%	3%	2%	T8	2%	4%	−1%	−2%	0%
O1	173%	29%	16%	15%	14%	O1	716%	123%	168%	128%	50%
O2	140%	30%	21%	13%	21%	O2	407%	41%	93%	82%	56%

## Discussion

### Summary of findings

This quasi-experimental study aimed to identify and quantify any changes that were present in brain EEG signals in the presence or absence of an audible pop following a HVLA manipulation at the T7 segment in healthy subjects. To our knowledge, this is the first study evaluating a pattern in brain wave activity between an audible and non-audible pop post-HVLA manipulation state.

### Interpretation of findings

The brainwave pattern observed in the non-audible pop group in our subjects resulted in an immediate decreased activity in the anteromedial right frontal lobe (AF4 and F4) with an increase in theta waves in the rest of the right and left frontal lobes, an increase in alpha and theta waves in the right parietal lobe, an increase in theta waves in the left parietal lobe, no change in right temporal lobe activity, a gross decrease in activity in the left temporal lobe (T7), and an increase in theta wave activity in the occipital lobes. These increases in Theta waves visible across most of the brain demonstrate a change in frontal lobe activity. An increase in theta wave activity correlates with a general relaxing effect, similar to a meditative state (Kumar & Bhuvaneswari, 2012). This finding supports the immediate generalized relaxation effect of HVLA manipulation without audible pop.

The brainwave pattern observed in the audible pop group was somewhat similar to that seen in the non-audible pop group, which also suggests an immediate relaxing effect in this group. There was an immediate generalized increase in theta wave activity in the bilateral frontal lobes, except for the most anterior portion of the right frontal lobe (AF4), where a general decrease in activity was observed. An increase in Theta wave activity in the bilateral parietal lobes was observed, a decrease in general activity in the left temporal lobe (T7), minimal change in activity in the right temporal lobe, and an increase in Theta wave activity bilaterally in the occipital lobes. Although both groups had similar increases in theta waves, the audible group had much larger changes when compared to the non-audible group, which could indicate that the presence of an audible pop has a directly affect the level of relaxation following an HVLA manipulation (Table 3).

### Comparison with other studies

Currently, there are no known patterns of responses in brain wave activity, measured with EEG, after an audible pop following an HVLA manipulation (Meyer et al., 2019). Meyer et al. (2019) report conflicting effects of HVLA manipulation on brain wave activity in their systematic review. It was identified that some studies report an overall increase in brain wave activity while others report a reduction of activity. No current studies in the last ten years using EEG while performing HVLA manipulation in healthy subjects could be identified, which limits the clinical discussion. The Bialosky et al. (2010) study in which heathy subjects underwent a dorsal thoracic spine thrust manipulation and the Moorman & Newell (2022) review were used to corralate our findings.

Audible sounds are perceived and interpreted in the brain by the primary auditory cortices of the temporal lobes. Therefore, it was hypothesized that there would be an increased activity under the T7 and T8 electrodes in the audible pop group compared to the non-audible pop group (Kliuchko et al., 2016). However, this hypothesis was not supported by our study results. Under the T8 electrode, which collects data from the right temporal lobe, there was a change of ± 5% across all waveforms in each group. Under the T7 electrode, which collects data from the left temporal lobe, there was a gross decrease in waveform activity between 7–34% in the non-audible group and a 69–95% decrease in the audible pop group; however, neither was statically significant (Table 3). According to Dunning et al. (2017), many HVLA manipulations have a unilateral effect. This might explain the differences identified in both temporal lobes.

The perception remains that the quality of an applied HVLA manipulation depends partially on the presence of the concurrently created audible pop. Either the anticipation, absence, or presence of audible sounds should alter brainwave activity. It was expected that there would be, at minimum, an increased activity under the parietal T7 and T8 electrodes in the audible group compared to the non-audible group (Kliuchko et al., 2016; Sparks et al., 2017). However, in contrast the results of our study demonstrate a downward inhibiting brainwave activity in the parietal lobes for non-audible and non-audible pop manipulations. This concurs with the findings of Sparks et al. (2017), who demonstrated a shift to a lower parietal wavelength (theta waves) following a thoracic HVLA manipulation in asymptomatic subjects.

There was a statistically significant increase in Theta band waves in the left parietal lobe (P7) in the audible group compared to the non-audible group. Before the manipulation, the P7 electrode was dominated by the Alpha band waves. This change in parietal brainwave activity could be due to the inhibiting central nervous system mechanisms that produce immediate hypoalgesia following a thrust manipulation (Meyer et al., 2019; Sparks et al., 2017). This collaborates with the findings of Sparks et al. (2017), who demonstrated a decrease in brainwave activity following their application of a thoracic thrust manipulation across the brain’s pain matrix. Our observed immediate changes after the HVLA manipulation for the Theta and Beta-L waves at P7 and P8 was hypothesized to be the direct effect of the HVLA manipulation. This observation conflicts with the findings of Bergamino et al. (2022), who reported on the belief that the audible pop was directly related to the effectiveness of an HVLA manipulation. Although we observed increased occipital lobe brainwave activity in both groups, this might not reflect any personal believes in the subjects regarding the success of an HVLA and audible sounds.

One has to consider that changes in brainwave activity throughout the brain could result from normal psychosocial response to touch, which previously has been related to a subjective increase in psychosocial well-being (Morita et al., 2018). Our results demonstrated a significant increase in Beta-L band wave activity in the right parietal lobe (P8) in the audible group compared to the non-audible group. Interestingly, this pattern of increase was more substantial in the right parietal lobe, which concurs with the findings of Morita et al. (2018). They reported a significant increase in brainwave activity in the right parietal lobe following hand placement on the thoracic spine.

A shift in activity at the left occipital lobe was demonstrated after the HVLA manipulation (O1) in the audible group compared to the non-audible group. There is currently no clear explanation for what mechanisms related to the audible pop would result in this unilateral change in activity in the left occipital lobe, as the intervention was applied bilaterally at the transverse processes of T7 with eyes closed. A statistically significant change between both groups with increased Beta-H band wave activity shows an increase in overall brain activity in this area. Baseline neurological activity in the occipital lobes is normally dominated by Alpha band wave activity, so a shift to the Beta-H band wave could indicate an increase in activity to levels commonly associated with increased mental activity and might be a representation of subject analysis that something good happened during the HVLA manipulation (Kumar & Bhuvaneswari, 2012).

The success of any therapeutic intervention could depend on the presence of placebo effect. The frontal lobe plays a key role in the placebo effect; a significant change in frontal lobe brain wave activity following an HVLA manipulation would be expected if the audible pop sound would carry such an effect in our subjects (Bakker & Miller, 2004; Wager & Atlas, 2015). Wager & Atlas (2015) demonstrated a downregulation in brainwave activity in the frontal lobe when subjects are exposed to a placebo effect, based on the fact that the changes in the frontal lobe in both audible and non-audible pop group did not vary it was concluded that that the audible pop did not produce a “placebo” response in our subjects.

### Strengths and limitations

This study helps to understand the response patterns in the brain wave activity measured with EEG, an HVLA manipulation, fulfilling the need proposed by the review of Meyer et al. (2019). This study was the first of its kind. Evaluating brainwave activity patterns between those experiencing an audible pop or non-audible pop HVLA thus contributes to the overall understanding of the effect of an HVLA manipulation. A limitation of this study was the relatively limited number of electrodes the Emotiv Epoc+ uses. A 14-lead EEG acquires fewer data points overall than a higher-lead EEG like a 20 or 50 lead EEG, making it more challenging to establish brain wave patterns and changes in band wave activities. The number of subjects in this study was limited and thus could lead to a Type II error, and extrapolation of the results to all healthy populations may not be realistic.

### Implication for future researchers

Future studies should further explore brain wave responses to HVLA manipulation in both symptomatic and asymptomatic individuals to see if similar patterns emerge, as found in this study. The clinical relevance of the audible pop needs to be explored further to determine if it has any meaningful clinical neurophysiological effects.

## Conclusion

This quasi-experimental repeated measure study found statistically significant changes in brain wave activity following an HVLA manipulation. Although both the non-audible and audible pop groups had significant changes, overall the audible pop group’s changes were more substantial. Overall, this study demonstrated an increase in Theta band wave activity across the frontal, occipital, and parietal lobes with minimal change or slight decline in activity in the temporal lobes. Similar overall brainwave changes were present between both groups, except for the bilateral parietal lobes and left occipital lobe, which had significant differences. This is the first study demonstrating differences in brainwave activity resulting from an audible pop during an HVLA manipulation of the thoracic spine; however, it still does not answer if the audible pop has any clinical significance for the patient’s health and functional outcomes.

##  Supplemental Information

10.7717/peerj.17622/supp-1 Supplemental Information 1 EEG data for pop and no pop

## Additional Information and Declarations

### Data Availability

The following information was supplied regarding data availability: The data is available in the Supplemental File.
